# Randomized controlled evaluation of the effect of music therapy with cognitive-behavioral therapy on social anxiety symptoms: Retraction

**DOI:** 10.1097/MD.0000000000019634

**Published:** 2020-03-13

The article “Randomized controlled evaluation of the effect of music therapy with cognitive-behavioral therapy on social anxiety symptoms”^[1]^ which appears in Volume 98, Issue 32 of *Medicine*, is being retracted over concerns regarding the validity of the statistical analysis. Authors were unable to provide the original dataset to address the concerns raised.
